# Modeling the environmental impacts of Asparagopsis as feed, a cow toilet and slurry acidification in two synthetic dairy farms

**DOI:** 10.1016/j.heliyon.2024.e29389

**Published:** 2024-04-13

**Authors:** René Méité, Lukas Bayer, Michael Martin, Barbara Amon, Sandra Uthes

**Affiliations:** a Leibniz Centre for Agricultural Landscape Research (ZALF), Eberswalder Straße 84, Müncheberg, Germany; b Albrecht Daniel Thaer-Institute of Agricultural and Horticultural Sciences, Department of Agricultural Economics, Humboldt University Berlin, Berlin, Germany; c IVL Swedish Environmental Research Institute, Valhallavägen 81, 114 28, Stockholm, Sweden; d KTH Royal Institute of Technology, Department of Sustainable Development, Environmental Science and Engineering, Teknikringen 10B, 114 28, Stockholm, Sweden; e Department Technology Assessment, Leibniz Institute for Agricultural Engineering and Bioeconomy (ATB), Potsdam, Germany and University of Zielona Góra, Poland; f System Dynamics Group, Department of Geography, University of Bergen, Bergen, Norway

**Keywords:** Life cycle assessment, Environmental impacts, Asparagopsis, Cow toilet, Slurry acidification, Agricultural landscapes

## Abstract

Intensive dairy farming, particularly enteric fermentation and manure management, is a major contributor to negative impacts on the local and global environment. A wide range of abatement measures has been proposed to reduce livestock-related emissions, yet the individual and combined effects of these innovations are often unknown. In this study, we performed an attributional life cycle assessment of three innovative measures modeled in two synthetic German dairy farm systems: Feeding of the seaweed *Asparagopsis*, installing an in-house cow toilet system, and performing on-field slurry acidification. These measures were modeled both individually and in combination to account for single and cumulative effects and compared to a reference scenario under current practices. Our results showed that feeding high levels of *Asparagopsis* and the combination of all three measures were most effective at reducing global warming potential (20–30 %), while only the latter mitigated eutrophication (6–9%) and acidification potential (14–17 %). The cow toilet required additional adapted manure management (separated storage and injection of urine) to effectively reduce eutrophication (8–10 %) and acidification potential (19–23 %) and to decrease global warming potential (3–4%) and abiotic depletion (4–5%). Slurry acidification slightly affected all considered environmental impact categories. All three measures involved trade-offs, either between LCA impact categories (global warming potential vs. abiotic depletion), the location of impacts (off- vs. on-farm), or the emission reduction in individual gases (ammonia vs. nitrous oxide). Measure combinations could compensate for the observed trade-offs. Our study highlights the potential of novel abatement measures but also shows the interdependencies of measures in different stages. This calls for a revisiting of current priorities in funding and legislation, which often focus on single objectives and measures (e.g. ammonia reduction) toward the preferential use of measures that are effective without driving trade-offs or improving resource efficiency.

## 1 Introduction

To curb climate change and to keep global warming below the 2 °C target compared to preindustrial levels, as agreed in the Paris Agreement, a transformation of social and economic practices and the integration of cost-effective measures to prevent, mitigate and reduce emissions are recommended in all sectors [1]. Agriculture is an important contributor to negative impacts on the environment, such as global warming, eutrophication and acidification, causing detrimental impacts at the local and global levels [2]. The agricultural sector in the European Union (EU) is responsible for 90 % of its total ammonia emissions (NH_3_), which mainly occur from manure management and the application of mineral fertilizers. Moreover, agriculture contributes to 10 % of total greenhouse gas (GWP) emissions, with methane emissions from enteric fermentation (enteric CH_4_) of livestock and nitrous oxide emissions from manure management and crop and grassland production [3]. Reducing livestock-related emissions has thus become an important political priority [4].

While sufficient and covered manure storage capacities as well as low-emission slurry field application techniques, e.g. by trailing hose or shoe, are already widely adopted, i.e. in Germany [5], other innovative and potentially more effective systems have been the subject of intensive research. For example, on dairy farms, novel measures that address environmental hotspots include feeding red macroalgae (*Asparagopsis*; seaweed) [6], the cow toilet [7] and on-field slurry acidification [8].

Feeding the red macroalgae *Asparagopsis* spp. can substantially reduce enteric methane emissions in dairy cows [[9], [10], [11], [12]] and cattle [13,14] due to its high bromoform content [6,15]. Other macroalgae tested show no effect on reducing methane emissions in trials [16,17]. A low inclusion level of 0.5 % of organic matter (OM) results in a moderate CH_4_ reduction and has no significant impact on milk yield [9], while a high inclusion level of 1 % of OM achieves a higher CH_4_ reduction but decreases milk yield [9,12,18]. No effects on milk yield are reported for canola oil steeped with *Asparagopsis armata* [10] or for liquid or pelleted formulations of *Asparagopsis armata* [11]. In cattle, CH_4_ emissions are reduced with inclusion levels of up to 0.5 % of OM without compromising meat quality or daily weight gain [6,14]. In response to the inclusion level of *Asparagopsis*, hydrogen production rises in dairy cows and cattle [9,13,14], while carbon dioxide production increases at high inclusion levels [9,14]. Seaweed inclusion of <1 % of dry matter intake (DMI) in livestock requires no further substitution of essential nutrients in the diet [19]. Concerns have been raised due to a probable decrease in long-term efficiency when constantly feeding *Asparagopsis* [6,20], and the palatability of seaweed since cows refused to eat the seaweed [18]. Another concern is the safe use of *Asparagopsis* since bromoform is recognized as a probable carcinogen for humans and animals, and long-term exposure may cause tumors [6] or affect the rumen wall [18]. Inclusion levels below 1 % of OM in dairy cows [9] and 0.5 % of OM in cattle [13,14] show no transfer of bromoform to milk, meat, blood, or feces in short-term studies. Conversely, other studies have detected bromoform in urine and milk [10,11,18], while after 17 days of feeding *Asparagopsis*, no traces were detected in milk, animal tissues or feces, regardless of the inclusion level in nonadapted dairy cows [18]. However, the concentrations of bromoform in the products were below the acceptable concentration limits. Naturally growing in temperate and tropical waters [21,22], *Asparagopsis* can be grown in land-based production systems [23], which reduces the risk of damage to the sea ecosystems due to a potentially intensified sea-based production, the dependence on seasonality [22], and enables controlled growth and shortened transport distances to farms. However, seaweed production is largely in the experimental phase, and only a few production plants are currently established in Europe [20,24].

The cow toilet system separates urine and feces by mechanically triggering the urinating reflex of the cows during additional concentrated feeding, thereby reducing ammonia emissions from housing [7,25]. A single toilet system can serve 25 dairy cows, collecting half of their excreted urine per day (15 L/cow) [26]. The separately collected urine contains a high ammonium nitrogen content with high plant availability and can thus substitute for mineral fertilizer [27]. The cow toilet system requires adapted manure management, including separated storage and land application of urine and slurry; otherwise, their remixing in a subsequent stage may reverse the prior emission reductions. The toilet system is listed in the Netherlands’ RAV list [28] as the best available technique for reducing ammonia emissions and has been available since 2021 [26].

Slurry treatment with sulfuric acid can reduce NH_3_, N_2_O and CH_4_ emissions during land application by decreasing the slurry pH [8,29,30] and decreasing N leaching [31,32], thereby increasing the availability of nutrients for plants and yields [32,33]. Current Danish legislation prescribes the addition of 3 kg of sulfuric acid per m³ of cattle slurry to ensure a minimum NH_3_ reduction of 25 % [34]. Slurry acidification requires additional liming, recommended 75 kg of CaCO_3_ (lime) per 1 L of sulfuric acid per tonne of slurry, which is required to avoid overacidification of soils [35]. Although acidification techniques have been used for more than a decade in the Netherlands and Belgium [36], they have not been widely adopted on farms in Germany [37].

All three measures have been reported to effectively reduce specific direct NH_3_ and GWP emissions in the respective management stage they address [[6], [7], [8]] but are either in the R&D phase or at a very low adoption level (in the EU). Therefore, reduction potentials are often only hypothesized or based on preliminary estimations due to the novelty of these measures, while their environmental impacts and associated trade-offs remain unclear. Thus, a holistic perspective is needed, encompassing the entire production system to assess their sustainability and to unveil potential trade-offs. In previous studies, life cycle assessment (LCA) has been used to assess the impacts of the dairy sector in different regions worldwide [[38], [39], [40], [41]], to compare management strategies [[42], [43], [44], [45], [46]], to assess the effects of management alignments and abatement measures [[47], [48], [49]], and to identify key drivers of emissions in dairy production [[50], [51], [52], [53]].

The objective of this study was to assess the potential for reducing the environmental impacts of the three abovementioned measures on two synthetic specialized German dairy farms for the product milk. The measures were modeled both individually and in combination to assess single and cumulative mitigation effects and highlight possible trade-offs.

## 2 Materials and methods

We employed an LCA, a widely applied method to holistically assess the impact of a product on the environment [54], following standardized protocols consisting of four phases: (1) Goal and scope definition, (2) inventory analysis, (3) impact assessment and (4) interpretation [55,56]. In addition to accounting for direct emissions, the LCA approach also comprises the burden of upstream processes, e.g. raw material extraction, energy generation and manufacturing of inputs, as well as the use phase until waste disposal.

### 2.1 Goal and scope

**Fig. 1:**
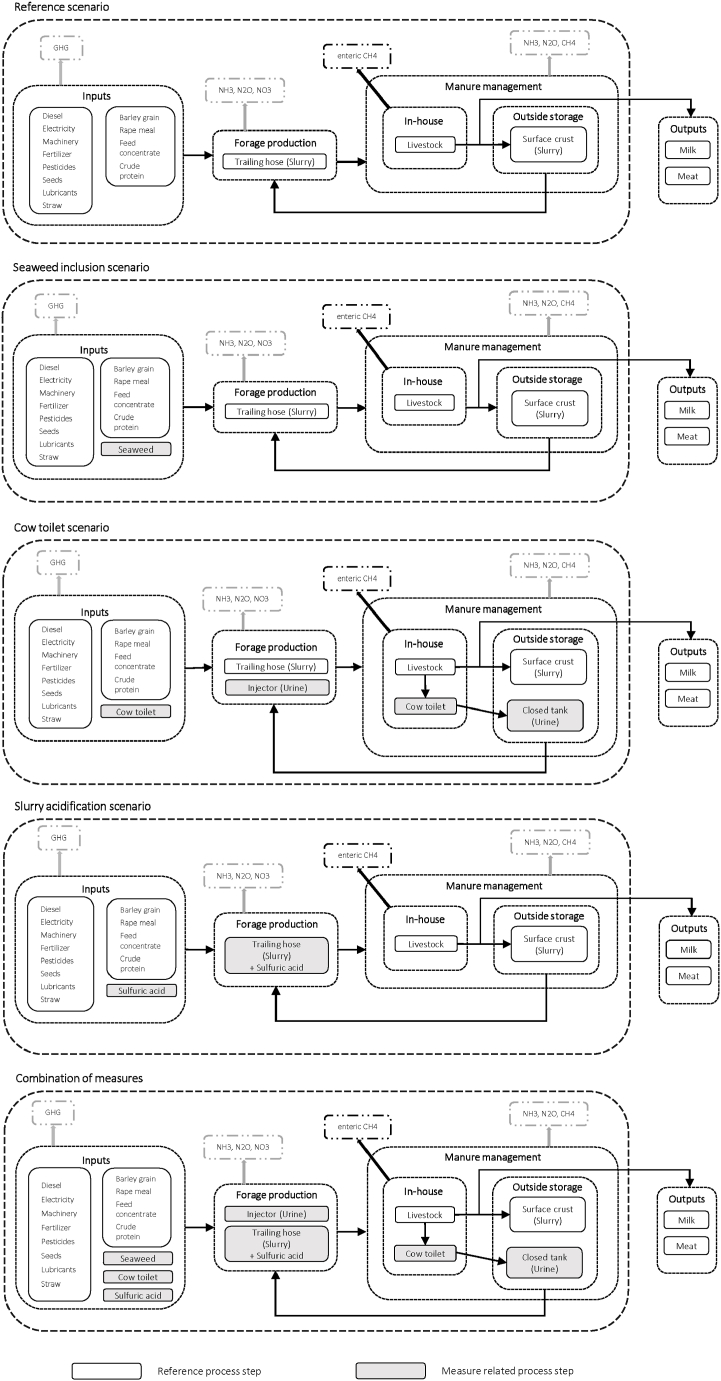
System boundaries of the scenarios (authors' own elaboration).

We chose the cradle-to-farm-gate system boundary (Fig. 1) to ensure comparability with the findings of other studies [38,48,49,57]. This included the production of required farm inputs for milk production, e.g. feed, fuel, fertilizer, energy and specific items (i.e. seaweed production, cow toilet and the acid tank) and of input feedstocks required for the abatement measures (i.e. seaweed and sulfuric acid). The farm milk production system included operations for forage production, feed purchase and slurry management (i.e. slurry storage and application; Fig. 1).

The functional unit was 1 kg of fat- and protein-corrected milk (FPCM). The FPCM was standardized to 4 % fat and 3.3 % true protein per kilogram [58]. The impact of milk as the main product and meat as a co-product was allocated according to the physical allocation proposed by the International Dairy Federation (allocation formula: AF_milk_ = 1–6.04 × 0.02; AF_meat_ = 1 - AF_milk_) [58]. This was done to check the results of the reference scenarios against the literature. To report on the reduction effects achieved, we did not account for the impacts of co-products (e.g. meat).

### 2.2 Case study areas

Our study focused on two synthetic conventional specialized dairy farms averaged on the basis of data from two German federated states, Lower Saxony (district Diepholz, DH) and Brandenburg (district Oder-Spree, OS), to account for the differences in the reduction potentials caused by the abatement measures. The farms differed in farm characteristics as a result of different political developments in East and West Germany before Germany's reunification in 1990 [59]. Compared with farms in Lower Saxony in Western Germany, farms in Brandenburg (Eastern Germany) are larger on average and have a greater livestock number, a larger field size, and larger machinery but also lower crop yields due to poorer soils [5].

### 2.3 Type of LCA

We performed an attributional LCA using openLCA v.1.11 [60] to model and calculate the environmental implications of different strategies for reducing direct emissions in livestock farming. To increase the comparability with the findings of other studies, we used the CML-IA baseline impact assessment method, which considers 11 impact categories [61]. We focused on four impact categories, namely, global warming potential (GWP; carbon dioxide equivalents, kg CO_2_e), eutrophication potential (EP; phosphate equivalent, g PO_4_^3−^e), acidification potential (AP; sulfur dioxide equivalent, g SO_2_e), and abiotic depletion (AD; fossil fuel depletion, MJ). These impact categories were chosen because they are influenced by the most relevant gaseous emissions in the agricultural context, i.e. ammonia (NH_3_), methane (CH_4_), nitrous oxide (N_2_O), carbon dioxide (CO_2_), and leaching (NO_3_), and thus are sensitive to changes caused by the analyzed abatement measures. These impact categories have also been employed in previous studies [38,44,48,49,57]. The conversion factors used to calculate the global warming potential over a 100-year time horizon were 28 for CH_4_ and 265 for N_2_O [61,62], while for the eutrophication potential, we used the conversion factor of 0.35 for NH_3_ [61].

### 2.4 Inventory analysis

Structural farm data were derived from the Integrated Administration and Control System (IACS; year 2017) for the regions Oder-Spree (OS) in Brandenburg and Diepholz (DH) in Lower Saxony (Table 1) to create two synthetic conventional specialized dairy farms [63]. We considered only farms with a ratio of ≥0.45 dairy cows per hectare of land to ensure that only truly specialized dairy milk-producing farms were included. Farms less than 5 ha and outliers were excluded, resulting in 8 and 214 farms for Oder-Spree and Diepholz, respectively, which could be averaged (Table 1).

**Table 1:** Main characteristics of the two synthetic specialized dairy farm systems in Oder-Spree (Brandenburg) and Diepholz (Lower Saxony) (authors’ own elaboration).

Parameter	Unit	Oder-Spree	Diepholz	Source
**milk production**				
number of farms	n	8	214	IACS, year 2017
number of dairy cows	n	666	121	IACS, year 2017
number of heifers	n	361	134	IACS, year 2017
milk yield (avg)	kg FPCM^a^	10,005	9,945	Dairy Control Associations
milk yield (sd)	kg FPCM^a^	725	1,143	Dairy Control Associations
milk yield (min)	kg FPCM^a^	8,672	8,073	Dairy Control Associations
milk yield (max)	kg FPCM^a^	11,355	12,384	Dairy Control Associations
milk yield (assumed)	kg FPCM^a^	10,000	10,000	Assumption
annual milk yield (assumed)	kg FPCM^a^	6,660,000	1,210,000	Assumption
**feed composition**				
forage	% total DM	71	58	[64]
feed concentrate	% total DM	29	42	[64]
**farm land**				
total land size	ha	806	111	IACS, year 2017
maize silage	ha	293	43	IACS, year 2017
alfalfa silage	ha	72	0	IACS, year 2017
grass silage	ha	54	10	IACS, year 2017
grass production	ha	73	33	IACS, year 2017
**cultivated forage**				
maize silage	% of total ha	36	38	[64]
alfalfa silage	% of total ha	9	0	[64]
grass silage	% of total ha	7	9	[64]
grass production	% of total ha	9	30	[64]
**crop yield**				
maize yield	t FM/ha	34.6^b^	44.1^b^	Federal and state statistical offices, 2022
alfalfa silage yield	t FM/ha	14.8	–	[66]; expert
grass silage yield	t FM/ha	15.9	30	[66]; expert
grass yield	t FM/ha	11.9	14.2	[66]; expert
**fertilization**				
maize silage	kg N/ha	162	170	[67,105]
alfalfa slage	kg N/ha	40 + 40^c^	–	[67]
grass silage	kg N/ha	80	170	[67]
grass production	kg N/ha	80	170	[67]
total mineral fertilizer	kg N/ha	53	55	[66]; expert
**further characteristics**				
soil yield level	SQR	low	medium	[65]
machinery power	kW	233	83	assumption based on field size
farm-to-field distance	km	15	5	assumption based on field size

Abbreviations: DM - dry matter, SQR - soil quality rating, IACS - Integrated Administration and Control System, avg - average, sd - standard deviation, min - minimum value, max - maximum value.

a FPCM - fat- and protein corrected milk.

b average of the years 2016–2018.

c 40 kg N/ha from N fixation by legumes (i.e. alfalfa).

We considered two cow categories, i.e. mature dairy cows, including the dry period and heifers (≤2 years), to account for the different amounts of feed intake, excreta and emissions in the different development stages (Table 1).

The feed composition of the forage and feed concentrate was calculated with the FAO Feed Ration Calculator [64] considering the livestock number of each farm and the available share of the farms’ forage production based on the IACS data (Table 1). Feed concentrates consisting of barley grain, rape meal, protein feed and mineral supplements were modeled as feed purchases and thus cannot be directly influenced by the farm management. Protein feed and mineral supplements were adopted from the Ecoinvent process and assumed to be constant in both synthetic farms.

The soil yield levels were obtained from Müller et al. [65]. Missing data for field operations, e.g. farm-to-field distance, machine size and power, amount of mineral fertilizer, diesel consumption, number of passes, number of cuts and forage yields, were obtained from the literature [66] and aligned with experts to account for regional specificity (Table 1; not all the data are shown).

The data for the amounts and nutrient contents of the excreta were adapted to the milk yield and age class of the cows, and the values of nitrogen requirements for the amount of applicable nitrogen fertilizer were derived from the German fertilizer ordinance [67] and aligned with crop yields, grassland use intensity (e.g. number of cuts) and soil yield level (Table 1). For alfalfa silage grown in Oder-Spree, we considered the nitrogen supply through N fixation [67]. The sum of the applied organic fertilizer agreed with the current fertilization legislation [67].

Emissions from enteric CH_4_, NH_3_, direct and indirect N_2_O emissions from manure management (in-house and outside storage), indirect N_2_O emissions from forage production and land applied lime were calculated using the IPCC inventory software v2.69 tier 1, assuming an average temperature of 11 °C [68]. NH_3_ emissions from loose housing were taken from Vos et al. [69]. For the calculation of NH_3_ and direct N_2_O emissions from the different land application techniques, we used the emission factors (EFs) for NH_3_ from broadcast slurry [69] and for N_2_O from organic fertilizer [70] as the basis and then recalculated changes in emissions for the trailing hose and slurry injection, applying the reduction potentials provided by Emmerling et al. [30] to account for possible pollution swapping effects (Table 2). For mineral fertilization, EFs for NH_3_ and N_2_O were considered, as reported by Vos et al. [69].

**Table 2:** Direct reduction effects and further impacts of the abatement measures (Asparagopsis as feed, cow toilet and slurry acidification) (authors’ own elaboration).

Stage	Abatement measure	Reference system	Emission gas	Reduction potential	Further adjustments	Source
feeding	seaweed (dairy cows) - low inclusion	conventional feed	CH_4_	-26 % at 0.5 % of OM		[9]
	seaweed (dairy cows) - high inclusion	conventional feed	CH_4_	-67 % at 1 % of OM	-12 % milk yield decrease	
	seaweed (heifer) - low inclusion	conventional feed	CH_4_	-45 % at 0.25 % of OM		[14]
	seaweed (heifer) - high inclusion	conventional feed	CH_4_	-68 % at 0.5 % of OM		
housing	cow toilet	loose housing	NH_3_	-40 %	separated storage and field application of urine and slurry	[28]
land application	trailing hose	broadcast application	NH_3_	-33 %		[30]^,a^
			N_2_O	+25 %		
	injection	broadcast application	NH_3_	-61 %		
			N_2_O	+19^a^ %		
	slurry acidification (trailing hose)	trailing hose	NH_3_	-45.7 %	additional liming; yield increase	[29]
			N_2_O	-21 %		[30]
			NO_3_	-18 %		[31]

Abbreviations: OM - organic matter intake as feed.

For the modeling in openLCA, we used the Ecoinvent database v3.8 and aligned available processes according to the derived data (Table 1). These included housing and liquid manure storage operations (e.g. energy) and straw addition. Elementary flows of the processes were adopted if no other data were available.

### 2.5 Assumptions

We considered the total livestock population in 2017 (Table 1) to maintain differences between the farms and therefore refrained from applying a culling or birth rate. Milk yields were derived from the Dairy Control Associations of the federate state regions Brandenburg and Lower Saxony and showed little difference between the regions; thus, they were assumed to be 10,000 kg FPCM per cow and year in both farms (Table 1). The housing type, storage facility and slurry application technique used were assumed to comply with the applicable legislation (Table 1). Regarding the fertilization of forage, we assumed that slurry is used as much as technically feasible for the respective forage type, considering possible restrictions for application, such as crop height or high damage due to the heavy weight of machines. Thus, the final fertilizer applied to the maize and grass silage was mineral fertilizer. We obtained tractor data according to the machine power used in the regions (Table 1) from typical machinery manufacturers and converted the machine weight and fuel consumption to other field activities. For comparison with the results of other studies, all impacts were attributed to 1 kg FPCM.

### 2.6 Scenarios

#### References

2.6.1

For the reference scenarios, livestock was assumed to be kept in a loose housing system (without grazing) on farms with slurry stored in tanks with a natural surface crust layer, and land application done by trailing hose without lime application. The farm data used are presented in Table 1.

#### 2.6.2 Feeding Asparagopsis

Seaweed inclusion was modeled in two subscenarios: (i) A low inclusion level (*SW low*, 0.5 % OM for dairy cows and 0.25 % of OM for heifers) and (ii) a high inclusion level (*SW high*, 1 % OM for dairy cows and 0.5 % of OM for heifers; Fig. 1), considering the specific CH_4_ reduction effects of the different animal stages and their impacts on milk yield (Table 2). The increase in hydrogen production reported in dairy cows and steers in response to seaweed inclusion was not considered [9,14].

Seaweed was assumed to be produced in a land-based system in Sweden (Lysekil) [23]. The LCA model of seaweed production was remodeled [23] and aligned to connect the production systems in openLCA using physical allocation for thermal energy provision. Energy provisioning for seaweed production was based on the Swedish energy mix [23]. Dried seaweed was assumed to be transported to German dairy farms by lorries (Fig. 1). Input and output data of the seaweed production system are given in Table SI6.

#### 2.6.3 Cow toilet

Data on the toilet system were obtained from the manufacturer [26]. We considered the required production of steel, rubber, a pump and energy. We assumed that only dairy cows use the toilet, while heifers continued to produce unseparated slurry. In total, the cow toilet collects between 20 and 30 % of the total excreted urine (OS: 29 %; DH: 23 %). We used the reduction potential (Table 2) stated in the RAV list [28], which was confirmed by a previous study [25]. We constructed two subscenarios, which we applied to both synthetic farms (Fig. 1). In the first cow toilet scenario (*CT1*), the cow toilet was implemented in the housing system without further adaptations. In the second cow toilet scenario (*CT2*), separate urine and slurry management was adopted. Urine was stored in a closed container and separated from the remaining excreta (slurry), assuming that no emissions occurred. Due to the high nutrient concentration in urine and to avoid corrosion of plants, field application of urine was assumed to be performed with an injector [26], irrespective of the forage type. The remaining excreta (nonseparated urine and feces) can be handled and thus, was modeled as slurry, stored in slurry tanks and applied by a trailing hose [26], as in the reference. Emissions from the stored slurry fraction were adapted to the remaining amount of slurry after the separation of urine. Nitrogen fertilization was adjusted according to the different loads of nitrogen in the urine and slurry (urine: 5.4 kg N/m³; slurry: 2.9–3.5 kg N/m³) [71], using the same maximum N fertilization values as those for the reference scenario (Table 1). Mineral fertilizer was assumed to be completely replaced by urine due to its high mineral fertilizer equivalent [27]. By substituting mineral fertilizer, the extraction and processing of inorganic N and P were also avoided.

#### 2.6.4 Slurry acidification (on-field)

Slurry acidification (*AS*) was assumed to be implemented only in the forage production stage and applied by trailing hose (Fig. 1). Sulfuric acid was modeled as a co-product from the desulfurization of natural gas and crude oil production in Hamburg (Germany). For the transport and use of acid, a tank with a capacity of 1000 L was used. We calculated the recommended dose of acid for cattle slurry required to avoid overacidification of soils (75 kg CaCO_3_ per 1 L acid) [35] based on the information given by the manufacturer [72], resulting in 1.5 L acid per m³ cattle slurry and 112 kg CaCO_3_ lime per 1.5 L acid. Maize silage yields were assumed to increase by 5 % [32], and grassland forage yields by 30 % [33,73], while total applied N amounts were assumed to remain the same as in the reference scenario (Table 1). NH_3_ and N_2_O reduction levels were derived from Nyameasem, Zutz et al. [29] and Emmerling, Krein et al. [30], respectively, while the percentage reduction of N leaching [31] was considered for forage of which Ecovinvent provided data on leaching, i.e. maize silage, alfalfa silage and grass silage. CH_4_ and CO_2_ emissions reduction effects were not applied since the Ecoinvent processes provided no suitable entries as a reference. The increased availability of sulfur for plants through the addition of sulfuric acid was not considered [74]. The emissions of lime were calculated with the IPCC inventory software v2.69.

#### 2.6.5 Measure combination

In a combined scenario (*CM*), we assumed that the three abatement measures with the highest possible direct reduction potential (*SW high, CT2, AS*) were applied together (Fig. 1) to assess cumulative effects. All the procedures were performed as described before. The increased crop yields and required amounts of acid and lime due to slurry acidification were considered as in scenario *AS*, while the acidified slurry quantity applied in *CM* was lower than that applied in *AS*, resulting from the separation into urine and slurry by the cow toilet.

## 3 Results and discussion

### 3.1 Reference scenarios

The results of the reference scenario are presented in Table 3 (absolute values) and in the supplementary information Table SI1 (relative values). The environmental impacts of the two farms in Oder-Spree (OS) and Diepholz (DH) were within the European average of dairy farms (Table 3) and were comparable to those of other cradle-to-farm gate LCA studies in conventional dairy production [48,49,53,57,75]. Overall, the OS farm type had lower impacts on all categories (Table 3). Consequently, the allocation between milk and meat as a co-product (beef credit) also resulted in a lower GWP in OS farm (Table 3).

**Table 3:** Comparison of results of the reference scenarios of this study with other cradle-to-farm gate LCA studies of conventional milk production per produced milk (authors’ own elaboration).

Source	FU	Assessment method	Allocation	AD [MJ]	AP [g SO_2_e]	EP [g PO_4_^3−^e]	GWP [kg CO_2_e]	Country
Oder-Spree	FPCM	CML	milk & meat	4.03	8.96	4.47	1.01	Germany
							0.89∗	
Diepholz	FPCM	CML	milk & meat	4.85	10.83	6.06	1.26	Germany
							1.11∗	
[38]	ECM	TIPI-CAL/IPCC 2007	milk & meat				1.10–1.4	Germany
							0.98–1.3∗	Germany
[44]	ECM	IPCC Tier 2	milk & meat				1.18	Germany
							1.15∗	
[51]	ECM	IPCC Tier 2, 3	none				1.08	Germany
[76]	FPCM						1.17	Germany
[48]	ECM		milk & meat	3.71	18.06	7.69	1.32	Germany
[57]	not given		milk & meat	2.7	19	7.5	1.3	Germany
[49]	FPCM	ReCipe 2016	milk & meat	2.53	6.8		0.97	Ireland
[106]	ECM	IPCC 1996 Tier 1	milk & meat				1.50	Ireland
							1.3∗	
[107]	ECM	IPCC 1996, 1997, 2000 Tier 1	none				0.76–1.26	Sweden
[75]	FPCM	IPCC 2006	milk, meat, grain		9.5		1.4	Netherlands
[108]	FPCM	ReCiPe	milk, meat, grain				1.30–1.32	Italy
[41]	ECM		milk & meat				1.12–1.16	USA
[53]	FPCM	IFSM	milk & meat	2.71			0.99	USA
[40]	FPCM	IFSM	milk & meat	2.48			1.01	USA

Abbreviations: AD - abiotic depletion, AP - acidification potential, EP - eutrophication potential, GWP - global warming potential, MEP - marine eutrophication potential, FEP - freshwater eutrophication potential, TAP - terrestrial acidification potential, ECM - energy-corrected milk, FPCM - fat and protein-corrected milk, FU - functional unit.

∗ Impact after deduction of beef credit.

**Fig. 2:**
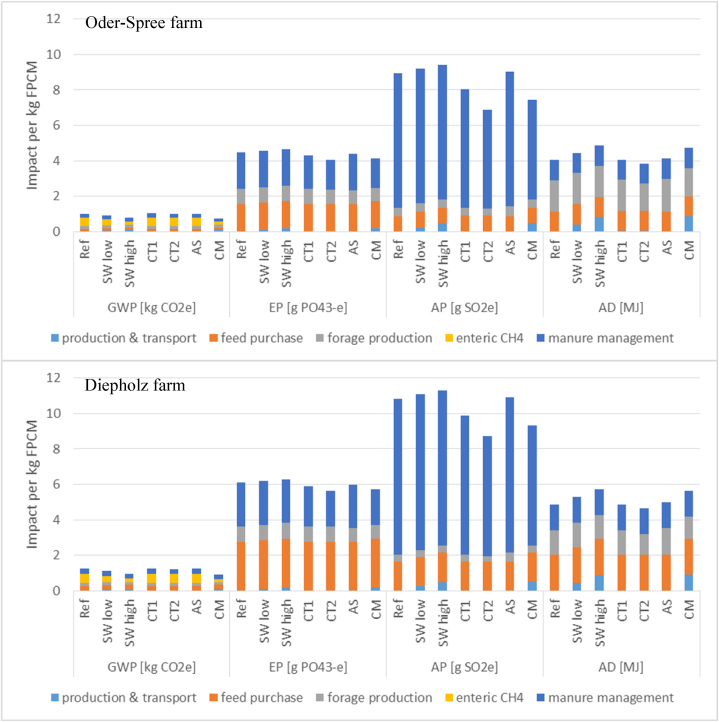
Scenarios results of the environmental impacts in Oder-Spree (Brandenburg) and Diepholz (Lower Saxony) differentiated by management stages; production & transport refers to measure-related inputs (authors' own elaboration).

For a further breakdown of impacts (Fig. 2), we distinguished five stages: (i) Production and transport of required inputs for abatement measures, i.e. production of dried seaweed, sulfuric acid and lime; (ii) feed purchase; (iii) forage production; (iv) enteric CH_4_; and (v) manure management, which included emissions from in-house and outside slurry storage. Feed purchases remained unchanged in the scenarios since the above measures did not influence this stage; thus, they were analyzed only in general terms.

Differences between the two farms in the reference scenario were due to differences in cow herd composition, which resulted in higher enteric CH_4_ concentrations and higher emissions from manure management in DH. Moreover, the machinery size, field size, amount of land applied slurry and cultivated forage types and yields, thus affecting the efficiency of producing forage and the amount of required compensation through feed purchase (Table 1), had greater impacts in DH (Table 4; Table SI1).

The **global warming potential** (GWP) in our LCA was strongly affected by enteric CH_4_ (OS: 46 %; DH: 44 %; Table 4; Table SI1). Literature-based contributions of CH_4_ ranged between 35 % and >80 % [44,48,49,76]. Together, the feed and forage provisions caused approximately 33 % of the GWP in both farm types, followed by the manure management treatment (23 %; Table 4; Table SI1), which agreed with the results from the literature [51,76]. For forage production, Ecoinvent provided no data on CH_4_ emissions; thus, fertilization had no influence on direct CH_4_ in our study. In both farms, N_2_O contributed less than 10 % to the GWP. The N_2_O emissions of the GWP from manure management accounted for less than 5 %, and those from fertilization in forage production were less than 4 % (Table 5; Table SI3), which were similar to the values found by Zehetmeier et al. [76].

The **eutrophication potential** (EP) was strongly affected by feed purchase and forage production (OS: 54 %; DH: 59 %) and manure management (OS: 47 %; DH: 40 %), with nearly equal shares of emissions originating from in-house and outside slurry storage (Table 4; Table SI1). The share of NH_3_ in EP was strongly influenced by fertilization in forage production (OS: 27 %; DH: 29 %) and manure management from outside storage (OS: 23 %; DH: 20 %), while in-house NH_3_ emissions contributed less to EP (OS: 13 %; DH: 10 %; Table 5; Table SI3). These contributions of NH_3_ to EP were comparable to the broadly ranging results in the literature [48,57], confirming that these emissions mainly occurred during manure storage and crop production [48].

The **acidification potential** (AP) in our LCA was also strongly influenced by manure management, with the highest share originating from outside storage (OS: 53 %; DH: 52 %), followed by the in-house stage (OS: 32 %; DH: 30 %; Table 4; Table SI1). The contribution of feed and forage provision to AP was moderate (OS: 15 %; DH: 19 %), with the larger share originating from feed purchase (Table 4; Table SI1). The literature shows that AP is strongly influenced by NH_3_ emissions from manure storage (>50 %) and forage production (11–40 %) [48], while up to 80 % of the NH_3_ in AP is due to NH_3_ volatilization from fertilization [57].

The **abiotic depletion potential** (AD) of both farms can be attributed mainly to feed purchase and forage production (approximately 70 %). However, in the OS farm, the largest share originated from forage production (OS: 44 %), while in the DH farm, the largest share originated from feed purchase (DH: 42 %; Table 4; Table SI1). Manure management accounted for approximately 30 % of AD (OS: 28 %; DH: 30 %), with a negligible share of impacts from outside storage (<0.01 %). These values were comparable to those of other studies, which reported that the contribution of AD to on- and off-farm activities can range between 17 % and more than 70 %, mainly occurring from commercial feed and fertilizer production [48,49].

### 3.2 Abatement scenarios

Table 4 presents the scenario results (absolute values) for each management stage, while Table 5 shows the contributions of CH_4_ and N_2_O to the GWP and of NH_3_ to the EP. The relative changes and contributions (percentage values) can be found in Tables SI1-SI5 in the supplementary information.

**Table 4:** Environmental impacts of the scenarios differentiated by management stages and expressed per kg of fat- and protein corrected milk (FPCM) for Oder-Spree and Diepholz (authors’ own elaboration).

Region	Impact category	Scenario	Production &transport	Feed purchase	Forage production	Enteric CH_4_	In-house	Outside storage	Total
**Oder-Spree**	GWP [kg CO_2_e]	Ref	0.000	0.149	0.166	0.465	0.100	0.134	1.014
		SW low	0.036	0.149	0.166	0.329	0.100	0.134	0.914
		SW high	0.071	0.149	0.166	0.161	0.100	0.134	0.781
		CT1	0.003	0.149	0.166	0.465	0.100	0.134	1.018
		CT2	0.003	0.149	0.157	0.465	0.100	0.104	0.978
		AS	0.000	0.149	0.167	0.465	0.100	0.134	1.014
		CM	0.075	0.149	0.159	0.161	0.100	0.104	0.748
	EP [g PO_4_^3−^e]	Ref	0.000	1.545	0.854	0.000	0.992	1.095	4.486
		SW low	0.087	1.545	0.854	0.000	0.992	1.095	4.573
		SW high	0.173	1.545	0.854	0.000	0.992	1.095	4.659
		CT1	0.007	1.545	0.854	0.000	0.788	1.095	4.289
		CT2	0.007	1.545	0.839	0.000	0.788	0.851	4.029
		AS	0.000	1.545	0.762	0.000	0.992	1.095	4.394
		CM	0.180	1.545	0.748	0.000	0.788	0.851	4.112
	AP [g SO_2_e]	Ref	0.000	0.883	0.462	0.000	2.864	4.747	8.956
		SW low	0.230	0.883	0.462	0.000	2.864	4.747	9.186
		SW high	0.460	0.883	0.462	0.000	2.864	4.747	9.416
		CT1	0.011	0.883	0.462	0.000	1.931	4.747	8.035
		CT2	0.011	0.883	0.385	0.000	1.931	3.687	6.897
		AS	0.001	0.883	0.538	0.000	2.864	4.747	9.033
		CM	0.471	0.883	0.450	0.000	1.931	3.687	7.422
	AD [MJ]	Ref	0.000	1.127	1.768	0.000	1.135	0.005	4.035
		SW low	0.412	1.127	1.768	0.000	1.135	0.005	4.447
		SW high	0.824	1.127	1.768	0.000	1.135	0.005	4.858
		CT1	0.031	1.127	1.768	0.000	1.135	0.005	4.066
		CT2	0.031	1.127	1.546	0.000	1.135	0.004	3.842
		AS	0.001	1.127	1.865	0.000	1.135	0.005	4.130
		CM	0.856	1.127	1.606	0.000	1.135	0.004	4.727
**Diepholz**	GWP [kg CO_2_e]	Ref	0.000	0.262	0.162	0.548	0.128	0.159	1.259
		SW low	0.038	0.262	0.162	0.375	0.128	0.159	1.124
		SW high	0.076	0.262	0.162	0.178	0.128	0.159	0.965
		CT1	0.001	0.262	0.162	0.548	0.128	0.159	1.259
		CT2	0.001	0.262	0.152	0.548	0.128	0.129	1.219
		AS	0.001	0.262	0.159	0.548	0.128	0.159	1.256
		CM	0.078	0.262	0.146	0.178	0.128	0.129	0.919
	EP [g PO_4_^3−^e]	Ref	0.000	2.749	0.881	0.000	1.169	1.290	6.089
		SW low	0.093	2.749	0.881	0.000	1.169	1.290	6.182
		SW high	0.186	2.749	0.881	0.000	1.169	1.290	6.275
		CT1	0.001	2.749	0.881	0.000	0.966	1.290	5.886
		CT2	0.001	2.749	0.865	0.000	0.966	1.042	5.623
		AS	0.001	2.749	0.780	0.000	1.169	1.290	5.988
		CM	0.188	2.749	0.759	0.000	0.966	1.042	5.703
	AP [g SO_2_e]	Ref	0.000	1.642	0.389	0.000	3.214	5.589	10.834
		SW low	0.247	1.642	0.389	0.000	3.214	5.589	11.081
		SW high	0.493	1.642	0.389	0.000	3.214	5.589	11.327
		CT1	0.002	1.642	0.389	0.000	2.282	5.589	9.904
		CT2	0.002	1.642	0.311	0.000	2.282	4.515	8.752
		AS	0.005	1.642	0.491	0.000	3.214	5.589	10.936
		CM	0.501	1.642	0.398	0.000	2.282	4.515	9.332
	AD [MJ]	Ref	0.000	2.033	1.367	0.000	1.447	0.006	4.852
		SW low	0.440	2.033	1.367	0.000	1.447	0.006	5.292
		SW high	0.880	2.033	1.367	0.000	1.447	0.006	5.732
		CT1	0.006	2.033	1.367	0.000	1.447	0.006	4.858
		CT2	0.006	2.033	1.166	0.000	1.447	0.005	4.656
		AS	0.007	2.033	1.492	0.000	1.447	0.006	4.977
		CM	0.893	2.033	1.258	0.000	1.447	0.005	5.628

Abbreviations: **Scenarios:** Ref - reference scenario; SW low - low seaweed inclusion level; SW high - high seaweed inclusion level; CT1 - cow toilet alone; CT2 - cow toilet with adapted manure management; AS - slurry acidification during land application; CM - combination of the measures (SW high, CT2 and AS). **Impact categories:** AD - abiotic depletion; AP - acidification potential; EP - eutrophication potential; GWP - global warming potential.

**Table 5:** Contribution of enteric CH_4_, N_2_O on total GWP in kg CO_2_e, and NH_3_ on total EP in g PO_4_^3−^e per kg of fat- and protein corrected milk (FPCM) (authors’ own elaboration).

Region	Scenario	GWP	EP	CH_4_			Direct N_2_O		Indirect N_2_O		NH_3_		
		Total	Total	Enteric CH_4_	Outside storage	Forage production	Outside storage	Forage production	Outside storage	Forage production	In-house	Outside storage	Forage production
		[kg CO_2_e]	[g PO_4_^3−^e]	[kg CO_2_e]			[kg CO_2_e]		[kg CO_2_e]		[g PO_4_^3−^e]		
**Oder-Spree**	Ref	1.01	4.49	0.47	0.08	0	0.032	0.025	0.021	0.016	0.568	1.038	1.187
	SW low	0.91	4.57	0.33	0.08	0	0.032	0.025	0.021	0.016	0.568	1.038	1.187
	SW high	0.78	4.66	0.16	0.08	0	0.032	0.025	0.021	0.016	0.568	1.038	1.187
	CT1	1.02	4.29	0.47	0.08	0	0.032	0.025	0.021	0.016	0.364	1.038	1.187
	CT2	0.98	4.03	0.47	0.06	0	0.025	0.029	0.016	0.016	0.364	0.806	1.190
	AS	1.01	4.39	0.47	0.08	0	0.032	0.019	0.021	0.014	0.568	1.038	0.594
	CM	0.75	4.11	0.16	0.06	0	0.025	0.023	0.016	0.019	0.364	0.806	0.646
**Diepholz**	Ref	1.26	6.09	0.55	0.10	0	0.038	0.032	0.026	0.021	0.628	1.222	1.742
	SW low	1.12	6.18	0.37	0.10	0	0.038	0.032	0.026	0.021	0.628	1.222	1.742
	SW high	0.96	6.27	0.18	0.10	0	0.038	0.032	0.026	0.021	0.628	1.222	1.742
	CT1	1.26	5.89	0.55	0.10	0	0.038	0.032	0.026	0.021	0.424	1.222	1.742
	CT2	1.22	5.62	0.55	0.08	0	0.031	0.037	0.021	0.021	0.424	0.988	1.703
	AS	1.26	5.99	0.55	0.10	0	0.038	0.024	0.026	0.018	0.628	1.222	0.813
	CM	0.92	5.70	0.18	0.08	0	0.031	0.028	0.021	0.018	0.424	0.988	0.854

Abbreviations:

**Scenarios:** Ref - reference scenario; SW low - low seaweed inclusion level; SW high - high seaweed inclusion level; CT1 - cow toilet alone; CT2 - cow toilet with adapted manure management; AS - slurry acidification during land application; CM - combination of the measures (SW high, CT2 and AS).

**Impact categories:** AD - abiotic depletion; AP - acidification potential; EP - eutrophication potential; GWP - global warming potential.

#### 3.2.1 Feeding Asparagopsis

Compared to the reference scenario, the seaweed inclusion influenced only the production and transport stages as well as the enteric CH_4_ emissions. Feeding the entire livestock herd a low intake of seaweed (*SW low*) had a small positive effect on the GWP (approximately -10 %; Table 4; Table SI2) through decreasing enteric CH_4_ production by approximately one-third (Table 5; Table SI4), while the values of the other three impact categories slightly increased, except for AD (OS: 10 %; DH: 9 %; Table 4; Table SI2). The impact of transport on all impact categories was less than <1 % (Table 4; Table SI1).

A high inclusion level of seaweed (*SW high*) reduced the GWP more effectively—by approximately 23 % in both farms compared to the reference (Table 4; Table SI2), as enteric CH_4_ was strongly reduced by more than 65 % (Table 5; Table SI4), despite the assumed reduction in milk yield to 8800 kg/cow/year (Table 2). However, *SW high* required twice the amount of dried seaweed as *SW low*, and thus, the required seaweed production doubled the impacts on the other three impact categories as well as on transport (Table SI2). However, the contribution of transport to all impact categories remained less than 1 % (Table SI1).

To our knowledge, there were no LCA studies on feeding *Asparagopsis* to dairy cows. For beef cattle, supplementation of the feed ration with *Asparagopsis taxiformis* can reduce the GWP of the Australian beef sector by 1–4% by 2030, considering the projected increase in GWP in the sector [77]. LCA studies on other feed additives, e.g. 3-nitroxypropanol (3NOP) and nitrate, have focused on the reducing effects of GWP and enteric CH_4_ [[78], [79], [80]]. The reduction effect of a low seaweed inclusion (*SW low*) in our study (Table SI2) was comparable to the effects of 3NOP (GWP: <-12 %; CH_4_: <-38 %) [79], while the additive nitrate had only a marginal reduction effect (GWP: <-4%; CH_4_: -14 %) [79]. A high seaweed inclusion (*SW high*) achieved the highest reduction potential among the comparable additives for GWP and enteric CH_4_ (Table SI2; Table SI4). However, the results depended on the considered herd composition and the duration of feeding the additives. The production of 3NOP and nitrate accounts for 35–52 kg CO_2_e/kg of produced 3NOP and 0.67–1.76 kg CO_2_e/kg of produced nitrate [79,80]. Seaweed production was between these values, causing 9.4 kg CO_2_e/kg per kg of produced dried seaweed [23]. Results for further impact categories are required for a sound comparison of the impacts of different feed additives.

For a low seaweed inclusion (*SW low*), to feed the total herd, a total of 24.8 t for the OS farm and 4.8 t for the DH farm would be required per year. A high inclusion level (*SW high*) doubled these amounts. The seaweed production system is assumed to produce 12 kg of dried biomass per day [23] (4.38 t per year), which could approximately supply the DH farm at a low inclusion level. For a high inclusion level in OS farm, more than eleven seaweed production systems are needed. Thus, upscaling seaweed production to supply all German or EU dairy farms seems unrealistic, as the land-based production, and the consumables, energy, and infrastructure required, could increase land use, amplifying the competition with, e.g. crop production, compared to sea-based algae production. Calculations have shown that 34 to 173 t dry weight of *Asparagopsis* biomass are required daily to supply 1.15 million of feedlot cattle in Australia and achieve a CH_4_ reduction between 42 % and 98 % [22]. Supplying dairy and cattle products with seaweed globally was estimated to be infeasible, but costs may be a further barrier for widespread use on smaller farms [19] since land-based systems are associated with greater land use and operational costs than sea-based cultivations are [24]. For grazing ruminants, there is no solution to manage the inclusion of seaweed [22].

The location of dried seaweed production considerably impacted all LCA categories in our study. Seaweed transport from Australia by ship (distance approx. 25,000 km) would increase all impacts per kg FPCM, particularly those of AP and AD (Table SI5), compared to the transport from Sweden to German farms by lorry (distance approx. 1000 km). Thus, the supply from Australia aggravates the trade-off between local emission reduction (Europe) and increases negative impacts in other parts of the world. However, shortening the transport distance by producing seaweed closer to farms would instead increase the necessary transport of input feedstocks (e.g. seawater, salts, and nutrients) and thus increase the impact of seaweed production compared to production sites with direct access to seawater. Thus, land-based seaweed production systems are preferably built on coasts that are less influenced by tides or urban development.

Regarding the direct reduction effect on enteric CH_4_ in dairy cows, high inclusion levels of *Asparagopsis* led to the highest achievable reduction effect (-67 %; Table 2) among available feed additives (3NOP: <-40 %; nitrate: <-14 %) [79]. However, all three additives decrease milk yield and may adversely affect animal health [6,20,81]. In contrast, the additive Slow Release Urease achieves a lower enteric CH_4_ reduction (dairy cows: 14 %) [82] without affecting milk yield [83]. In addition to CH_4_ reduction, other feed additives, e.g. plant extracts, may also provide co-benefits, such as reducing NH_3_ emissions; however, these co-benefits have large uncertainties [84] and are thus not further discussed here.

#### 3.2.2 Cow toilet

The cow toilet alone (*CT1*) caused a slight reduction in the impact categories EP (OS: -4%; DH: -3%) and AP (OS: -10 %; DH: -9%) due to reduced in-house NH_3_ emissions and had small negative impacts on GWP and AD (<1 % in both farms; Table 4; Table SI2), which could be attributed to the additional energy demand required for the production and use of the toilet. In-house NH_3_ emissions were moderately reduced by one-third (Table 5; Table SI4) and were less than the estimated potential of 40 % (Table 2) since only dairy cows used the toilet, while heifers continued to excrete on the floor. For the entire manure management chain, only a relatively small NH_3_ reduction could be achieved (OS: -7%; DH: -6%; Table 5).

In *CT2*, adapted subsequent management had further positive impacts on all impact categories (Table SI2) compared to *CT1*. By adapting the storage and application of urine, the negative impacts on GWP and AD resulting in *CT1* were reversed in *CT2*. Less energy was required to mix a smaller volume of stored slurry, and less mineral fertilizer was produced by replacing it with urine (in total OS: 53; DH: 55 kg N; Table 1), reducing GWP and AD. These benefits exceeded the increased energy demand for the production and use of the toilet. The total NH_3_ reduction on the entire manure management chain reached approximately 14 % (OS: -16 %; DH: -13 %; Table 5).

The improvements for the EP and AP categories could be attributed to the in-house NH_3_ reduction (see results of *CT1*), while the separated and closed storage of urine improved the impacts of GWP, EP and AP due to reduced NH_3_, CH_4_ and N_2_O emissions by approximately one-fifth compared to the reference (Table 4) since urine storage was assumed to not evoke emissions. In forage production, EP was affected by the substitution of mineral fertilizer by urine applied with an injector. NH_3_ emissions slightly increased in OS and decreased in DH (Table 5). This was mainly due to the different shares of produced forage and amounts of substituted fertilizer between farms. Depending on the considered proportion of on-farm produced forage that is required as feed and the amount of mineral fertilizer replaced by urine, increased NH_3_ emissions are caused despite of urine injection as a low-emission technique since the NH_3_ and N_2_O emission factors of organic fertilizers (urine and slurry) are greater than those of mineral fertilizers [69,70]. Due to the pollution swapping caused by urine injection (Table 2), in both farms, direct and indirect N_2_O emissions increased by approximately 15 % and <3 %, respectively, compared to the reference values, thus negatively affecting GWP (Table 5; Table SI4). However, the adaptation of urine application is recommended since the overall reduction in GWP outperforms the increase in N_2_O from urine injection. With an increasing share of substituted mineral fertilizer by injected urine, on-farm emissions could further increase while reducing the off-farm impacts of production and transportation.

To our knowledge, LCA studies of cow toilet systems are unavailable. Thus, we compared our results to those of other in-house measures. For example, similar effects were reported for in-house solid–liquid separation, which reduced terrestrial acidification (TA) by more than 40 % and GWP by more than 85 % in cattle and pig production without causing pollution swapping [85]. Comparable results were found for in-house separation by a V-belt in fattening pigs, which included separating streams of urine and feces in covered storage and soil injection and reducing impacts on GWP, TA and particulate matter formation (PMF; an indicator that is also used to assess changes in ammonia emission) while saving mineral fertilizer [86]. Similarly, in our study, lower N_2_O emissions could be achieved through separated and covered storage, while lower NH_3_ emissions were achieved through injection compared to unseparated covered storage and slurry injection [86]. However, higher N leaching resulted during field application due to a greater N content in feces [86]. Fossil fuel depletion (a comparable indictor for abiotic depletion) only slightly increased since the avoided mineral fertilizer and additional required transport for both fractions (urine and feces) almost completely balanced each other [86]. Increasing impacts on GWP resulted mainly from N_2_O when switching from the injection of slurry (reference) to the injection of urine and feces [86]. Overall, however, the avoidance of mineral fertilizers compensates for pollution swapping effects.

Other in-house separation systems, e.g. grooved, perforated, or sloped surfaces, can reach comparable or greater NH_3_ reduction levels, approximately 35–70 % [[87], [88], [89]], and use only gravity to drain urine; thus, they are not reliant on additional energy in contrast to the cow toilet (Table 2). However, a disadvantage of these systems is that urine is contaminated, while the cow toilet offers the option to collect urine without contamination. Modeling has shown that by collecting approximately 80 % of the daily urine volume, NH_3_ can be reduced by 56 % [90]. Collecting higher volumes might technically not be feasible. Comparable approaches to cow toilet are in development [91].

The cow toilet seems to be a relatively costly solution, with approximately 1,000 € per animal, including investment and variable costs [92], but may provide further benefits for farmers, such as cost savings for mineral fertilizer and the ability to adapt fertilization to the needs of the plants.

#### 3.2.3 Slurry acidification (on-field)

Slurry acidification (*AS*) had only marginal reducing effects on all four impact categories in both farms, reducing EP by approximately -2% but increasing AP and AD by approximately 0.9 % and 2.5 %, respectively (Table 4; Table SI2). This was because *AS* was introduced only in the forage production stage. With a greater share of on-farm produced feed than feed purchased, the overall reduction effect could increase. The production and transport of lime and sulfuric acid contributed less than 1 % of the impacts (Table 4).

In the forage production stage, *AS* reduced EP (OS: -11 %; DH: -12 %; Table 6), resulting in an NH_3_ reduction of approximately 50 % from the applied slurry and 18 % from nitrate compared to the reference (Table 5; Table SI4). In contrast to the decreasing EP, the impacts of AP (OS: 17; DH: 26 %) and AD (OS: 6; DH: 9 %; Table 6) increased due to the production and application of sulfuric acid and lime (Table 4). Neglectable impacts occurred for GWP (Table 6) due to the reduction in N_2_O emissions by approximately one-third (Table 5), which exceeded the emissions from additional lime application (data not shown). Differences in emissions between the two farms could be attributed to the different amounts of applied slurry and thus the required amounts of acid and lime. Only in DH farm the share of production and transport of the total impact of forage production on AP was above >1 % (Table 6).

**Table 6:** Results of the environmental impacts of slurry acidification and the reference in forage production for Oder-Spree and Diepholz expressed per kg of fat- and protein corrected milk (FPCM) (authors’ own elaboration).

Region	Impact category	Ref forage	AS forage	Reduction effect∗	Share of production & transport
**Oder-Spree**	GWP [kg CO_2_e]	0.166	0.167	0.1	0.05 %
	EP [g PO_4_^3−^e]	0.854	0.762	-10.8	0.02 %
	AP [g SO_2_e]	0.462	0.538	16.6	0.12 %
	AD [MJ]	1.768	1.865	5.5	0.05 %
**Diepholz**	GWP [kg CO_2_e]	0.162	0.159	-1.8	0.43 %
	EP [g PO_4_^3−^e]	0.881	0.780	-11.5	0.19 %
	AP [g SO_2_e]	0.389	0.491	26.1	1.10 %
	AD [MJ]	1.367	1.492	9.1	0.48 %

Abbreviations:

**Scenarios:** Ref forage - forage production of reference scenario; AS forage - forage production of slurry acidification scenario.

**Impact categories:** AD - abiotic depletion; AP - acidification potential; EP - eutrophication potential; GWP - global warming potential.

∗ Negative values mean a reduction, positive values mean an increase of the specific impact.

Slurry acidification can potentially achieve a greater reduction in GWP when CH_4_ and CO_2_ mitigation are also considered [30]; however, this approach could not be included in this study. Meta-studies have also reported greater achievable NH_3_ reduction using sulfuric acid [30,93], which would have reduced the impact of EP to a greater extent. However, we decided to use a lower value resulting from a trial in which cattle slurry was applied on grassland by trailing hose under German conditions (Table 2).

A comparison of our results with the literature was hampered by varying functional units and system boundaries. Thus, we compared the results in terms of the direction of effects. Similar to our results, studies have shown that acidified field-applied pig slurry lowers eutrophication-related impacts, i.e. terrestrial eutrophication potential (TEP), while increasing GWP [94,95] due to emissions from the production and application of acid and lime compared to the reference (storage and application of untreated slurry) [[94], [95], [96]]. In-house acidifying slurry affects subsequent manure management during storage and field application and can further reduce EP [[94], [95], [96]] and AP [96]. However, the GWP increases [[94], [95], [96]].

In contrast, studies that use other functional units (e.g. 1000 kg of slurry or 1 kg of live weight pig) report a reduced GWP when assessing acidified land-applied slurry compared to the application of untreated slurry since on-farm emission reduction is greater than emissions from the production of acid and lime [97]. An increasing energy demand results from increased acid production and the required mixing of acid with slurry [96,97]. A decrease in AP is due to reduced emissions during storage and land application [96,97] and to the adjustment of the slurry quantity and resulting savings in mineral fertilizer [97]. In this study, the amounts of mineral fertilizer and sulfur were not adjusted. Instead, we considered these effects indirectly through increased forage yields. In addition, Beyer et al. reported that adjusting the sulfur content had only a negligible effect on the results [97].

In contrast to other field-level abatement measures, such as injection or incorporation, which achieve a high NH_3_ reduction at risk of increasing N_2_O emissions, slurry acidification provokes no pollution swapping while mitigating CH_4_ emissions [98]. Other slurry additives (e.g. biochar, urease and nitrification inhibitors) target specific gaseous emissions, e.g. NH_3_ or N_2_O, and achieve a lower emission reduction than sulfuric acid does [[98], [99], [100], [101]].

The investment costs of slurry acidification systems are approximately 100,000 € and approximately 0.35 €/L acid [74]; thus, slurry acidification is likely to be an appropriate technique for contractors to use. Additional income can be gained through a higher yield and decreased use of mineral fertilizer [74], while further costs are associated with additional lime.

#### 3.2.4 Measure combination

The measure combination (*CM*) of *SW high*, *CT2* and *AS* reduced the impacts of GWP, EP and AP (OS: GWP -26 %, EP -8%, AP -17 %; DH: GWP -27 %, EP -6%, AP -14 %), while AD increased (OS: 17 %; DH: 16 %) compared to the reference values (Table 4; Table SI2). The reduction in enteric CH_4_ caused by feeding seaweed contributed the most to the reduction in GWP by two-thirds, followed by the reduction by one-fifth of GWP in the stage of slurry storage through the separation of urine and slurry, compared to the reference (Table 5). The increase in AD can be mainly attributed to measure-related inputs, mainly the production of seaweed, which contributes by approximately 17 % to the total AD in both farms and cannot be offset by the substitution of mineral fertilizer by urine, collected with the cow toilet (Table 4). The decrease in EP and AP (Table 4) is driven by the in-house NH_3_ emission reduction through the use of the cow toilet (OS: -36 %; DH: -33 %) and from separated storage of urine and slurry (OS: -22 %; DH: -19 %) compared to the reference (Table 5; Table SI4). Increases in EP and AP, due to the production and transport of measure-related inputs (contribution of approx. 4 % and 6 % to total EP and AP, respectively; Table SI1), are compensated. The injection of urine and slurry acidification (*AS*) in forage production only plays a minor role in the overall emission reduction, as explained above, yet both techniques contribute to halving NH_3_ and moderately reducing total N_2_O emissions in both farms compared to the reference (Table 5; Table SI4). Overall, the share of transportation still accounts for less than 1 % of the total impacts.

In other studies, combination measures, e.g. in-house segregation, storage cover, low-emission application techniques, acidification and anaerobic fermentation, have been shown to reduce environmental impacts while avoiding pollution swapping and increasing nitrogen use efficiency [85]. However, including measures such as slurry incorporation or injection results in beneficial effects in reducing AP and EP but increases GWP [102].

Meta-studies that assessed on-farm NH_3_ and GWP emissions suggest that a great variety of combinations of measures successfully mitigate emissions in pig and cattle production [98,103,104]. However, combining measures that cause the same side effects increases the GWP, while combinations that exert opposite effects compensate for the detrimental effects and reduce overall on-farm emissions [98]. However, these meta-studies focused on direct emissions and neglected upstream processes.

### 3.3 Comparison of scenarios

The scenarios in which single measures were combined (*CT2* and *CM*) generally performed better than single measures alone (e.g. *SW low*, or *CT1*) since different management stages and gaseous emissions were addressed and thus compensated for the occurring trade-offs (Fig. 2).

*CM* realized the largest GWP reduction (Fig. 2) but caused the highest share of impacts on the GWP in the production and transportation stage among the scenarios due to the additional inputs needed. *SW high* caused the second highest reduction of GWP, while *SW low* resulted in a small reduction. *CT2* and *AS* avoided increasing the GWP while *CT1* marginally affected the GWP negatively through measure-related inputs (Fig. 2).

The greatest reductions in EP and AP were achieved in *CT2*, followed by CM, in which seaweed production decreased the reduction effect of EP and AP (Fig. 2). *CT1* slightly reduced in-house NH_3_ emissions, thus decreasing EP and AP (Fig. 2). *AS* caused the lowest reduction in EP but slightly increased AP producing and using acid and lime. However, when comparing the scenarios in the forage production stage, *AS* performed best in reducing NH_3_ and N_2_O emissions since mineral fertilizer was used in *AS*, which had lower emission factors than organic fertilizers (urine and slurry) used in *CT2* (Table 5). Both *SW low* and *SW high* increased the impacts of EP and AP (Fig. 2).

*SW high* caused the highest impact of AD among the scenarios, followed by *CM* (Fig. 2). Only *CT2* can compensate for the increase in AD as a result of substituting mineral fertilizer. *CT1* and *AS* only slightly changed AD (Fig. 2).

### 3.4 Trade-offs

Different trade-offs were identified:

(1) Between impact categories: Abatement measures decrease the targeted impact(s), e.g. GWP or EP, while increasing others, e.g. AD.
(2) Within a single impact category, a shift between off- and on-farm impacts can occur, e.g. GWP, affecting the location of emissions.
(3) Between different gases (pollution swapping), an abatement measure can mitigate one gas but increase the emissions of another, e.g. for NH_3_ and N_2_O.

Each scenario leads to at least one of the above trade-offs, which we illustrate. In the *SW low*, *SW high*, *CT1*, *AS* and *CM* scenarios, at least one impact category (e.g. GWP) decreased, while the others increased, e.g. AD (trade-off 1). Only *CT2* caused no trade-off 1 since increasing emissions in one stage (e.g. forage production) were compensated through, e.g. mineral fertilizer substitution (Table 4). Moreover, seaweed inclusion reduced the local on-farm GWP while increasing off-farm impacts at production sites (trade-off 2; Table 4). The opposite effect occurred when *CT2* reduced off-farm impacts through the avoidance of mineral fertilizer production while increasing on-farm emissions using organic fertilizers (trade-off 2; Table 5). In forage production in *CT2*, pollution swapping (trade-off 3) occurred, as injection reduced NH_3_ emissions but increased N_2_O emissions. *AS* caused no trade-off 3 (Table 5). Other LCA studies found similar trade-offs for other abatement measures such as slurry acidification or in-house solid-liquid separation [86,97].

Single measures can prevent only trade-offs if they are effective at reducing at least one gaseous emission without causing pollution swapping or requiring additional input materials. Combinations of measures can prevent or reduce trade-offs, even if a single measure in a bundle may negatively impact one management stage. Combinations that evoke the same trade-off aggravate the adverse effect.

Whether combinations of measures perform better also depends on the assumptions of the reference scenario. In this study, we intentionally chose permitted measures to demonstrate the additional reduction potentials of further measures and their trade-offs.

From a sustainability perspective, three strategic questions become apparent from our analysis:

First, should we promote technologies for local emission reduction that require additional inputs at the cost of increasing global emissions, or vice versa?

Second, which country should account for emission reductions and increases? Since countries must report their national emission reduction success to meet national and global emission targets, countries that produce items at the expense of increasing local impacts for reducing local impacts elsewhere need to compensate for this increase to meet their emission targets. Thus, global reduction accounting should be preferred to not drive inequalities between countries.

Third, how can further emission reduction be realized? The effort (e.g. work, time and costs) for further emission reduction beyond “the low hanging fruits” (cost-effective abatement measures) is increasing, while the additional reduction success decreases, which is a function of the diminishing marginal utility. A 100 % reduction is not technically feasible. Therefore, the prevention and mitigation of emissions should be favored over treating emissions with additional products.

## 4 Limitations

The robustness of the results of an LCA is reliant on the quality of the available data and often involves a number of assumptions that can lead to uncertainties. These uncertainties can be analyzed, e.g. by using sensitivity analysis to identify the influence of different parameters and methodological choices on the environmental performance. A limitation of this study was that no sensitivity analysis was performed. This was due to the fact that a number of different options were analyzed. To minimize uncertainties and to increase the robustness of our model, we created two regionalized synthetic farms based on data that represent the specific German conditions (Table 1). However, we recommend that future studies analyze the influence on the results of variations in the CH_4_ reduction effectiveness of feeding *Asparagopsis* in cows, the amount of urine collected by the cow toilet, and the effectiveness of emissions mitigation of applying acidified slurry to fields.

## 5 Conclusion

Our results showed that the considered innovative abatement measures for dairy farms (*Asparagopsis* as feed, cow toilet and on-field slurry acidification) can reduce environmental impacts to varying degrees and showed only slight differences in their effects between the two synthetic farms. However, all the scenarios involved trade-offs.

Among the scenarios, the combination of *CM* (*SW high*, *CT2*, and *AS*) achieved the greatest reduction in GWP while also decreasing EP and AP. *CT2* reduced EP and AP the most, without causing negative effects overall. Of the individual measures, *SW high* mitigated the GWP the most effectively but increased EP and AP. *CT1* led to a small reduction in EP and AP. *AS* included in forage production exerted only minor effects on the two dairy farm systems. In the forage production stage, *AS* achieved the greatest reduction in EP, yet this change caused a drastic increase in AP. Trade-offs occurred mostly between the reduction in impacts, e.g. GWP or EP, and increasing AD due to increased impacts of the production and use of the measures. The combination of measures could reduce and compensate for the trade-offs of individual measures since different management stages and emissions were addressed.

Our study highlights the interrelations between different abatement measures and shows how measure combinations can complement each other. As current guidelines for farmers often focus on measures to reduce the emission of single gases (e.g. ammonia gas), our results call for a revisiting of priorities in funding and legislation toward a more holistic perspective and system thinking that favors and supports measures along the manure management chain to reduce trade-offs and unwanted path dependencies. Given that none of the analyzed scenarios could fully offset trade-offs, measures that reduce more than one gas without driving pollution swapping while not requiring additional constant inputs should be prioritized. However, this needs to be negotiated with possible further trade-offs, such as increasing impacts elsewhere, costs, and recognizable benefits for farmers.

## Data availability statement

Data included in article/supp. material/referenced in article.

## CRediT authorship contribution statement

**René Méité:** Writing – review & editing, Writing – original draft, Visualization, Validation, Software, Resources, Project administration, Methodology, Investigation, Formal analysis, Data curation, Conceptualization. **Lukas Bayer:** Writing – review & editing, Validation, Software, Methodology. **Michael Martin:** Writing – review & editing, Validation, Software, Resources, Methodology. **Barbara Amon:** Writing – review & editing, Supervision. **Sandra Uthes:** Writing – review & editing, Supervision, Project administration, Funding acquisition, Data curation.

## Declaration of competing interest

The authors declare that they have no known competing financial interests or personal relationships that could have appeared to influence the work reported in this paper.
